# Exploring views of members of the public and policymakers on the acceptability of population level dietary and active-travel policies: a qualitative study

**DOI:** 10.1186/s12966-023-01465-7

**Published:** 2023-05-31

**Authors:** Z Toumpakari, S. Valerino-Perea, K. Willis, J. Adams, M. White, M. Vasiljevic, L. Ternent, J. Brown, M. P. Kelly, C. Bonell, S. Cummins, A Majeed, S. Anderson, T. Robinson, V. Araujo-Soares, J. Watson, I. Soulsby, D. Green, F. F. Sniehotta, R. Jago

**Affiliations:** 1 Centre for Exercise, Nutrition and Health Sciences, School for Policy Studies, University of Bristol, 8 Priory Road, Bristol, BS8 1TZ UK; 2 Population Health Sciences, Bristol Medical School, University of Bristol, Canynge Hall, 39 Whatley Road, Bristol, BS8 2PS UK; 3 MRC Epidemiology Unit, University of Cambridge, Cambridge, UK; 4 Fuse – Centre for Translational Research in Public Health, Newcastle, UK; 5 Department of Psychology, Durham University, Durham, UK; 6 Population Health Sciences Institute, Faculty of Medical Sciences, Newcastle University, Newcastle, UK; 7 Department of Behavioural Science and Health, University College London, London, UK; 8 SPECTRUM Consortium, London, UK; 9 Department of Public Health and Primary Care, University of Cambridge, Cambridge, UK; 10 Department of Public Health, Environments and Society, London School of Hygiene & Tropical Medicine, London, UK; 11 Department of Primary Care and Public Health, Imperial College London, London, W6 8RP UK; 12 The National Institute for Health Research, Applied Research Collaboration Northeast and North Cumbria (NIHR ARC NENC), St Nicholas’ Hospital, Newcastle Upon Tyne, Jubilee Road, Gosforth, NE3 3XT UK; 13 Faculty of Behavioural, Management and Social Sciences, Department of Health Technology and Services Research, University of Twente, Twente, The Netherlands; 14 South Gloucestershire Council, Badminton Road, Yate, Bristol, BS37 5AF UK; 15 Department for Public Health, Social and Preventive Medicine, Medical Faculty Mannheim, Heidelberg University, Heidelberg, Germany; 16 Applied Research Collaboration West (NIHR ARC West), The National Institute for Health Research, University Hospitals Bristol and Weston NHS Foundation Trust, Bristol, BS1 2NT UK

**Keywords:** Acceptability, Effectiveness, Fairness, Policy communication, Policy, Public health, Active travel, Diet

## Abstract

**Background:**

There is limited evidence on what shapes the acceptability of population level dietary and active-travel policies in England. This information would be useful in the decision-making process about which policies should be implemented and how to increase their effectiveness and sustainability. To fill this gap, we explored public and policymakers’ views about factors that influence public acceptability of dietary and active-travel policies and how to increase public acceptability for these policies.

**Methods:**

We conducted online, semi-structured interviews with 20 members of the public and 20 policymakers in England. A purposive sampling frame was used to recruit members of the public via a recruitment agency, based on age, sex, socioeconomic status and ethnicity. Policymakers were recruited from existing contacts within our research collaborations and via snowball sampling. We explored different dietary and active-travel policies that varied in their scope and focus. Interviews were transcribed verbatim and analysed using thematic reflexive analysis with both inductive and deductive coding.

**Results:**

We identified four themes that informed public acceptability of dietary and active-travel policies: (1) perceived policy effectiveness, i.e., policies that included believable mechanisms of action, addressed valued co-benefits and barriers to engage in the behaviour; (2) perceived policy fairness, i.e., policies that provided everyone with an opportunity to benefit (mentioned only by the public), equally considered the needs of various population subgroups and rewarded ‘healthy’ behaviours rather than only penalising ‘unhealthy’ behaviours; (3) communication of policies, i.e., policies that were visible and had consistent and positive messages from the media (mentioned only by policymakers) and (4) how to improve policy support, with the main suggestion being an integrated strategy addressing multiple aspects of these behaviours, inclusive policies that consider everyone’s needs and use of appropriate channels and messages in policy communication.

**Conclusions:**

Our findings highlight that members’ of the public and policymakers’ support for dietary and active-travel policies can be shaped by the perceived effectiveness, fairness and communication of policies and provide suggestions on how to improve policy support. This information can inform the design of acceptable policies but can also be used to help communicate existing and future policies to maximise their adoption and sustainability.

**Supplementary Information:**

The online version contains supplementary material available at 10.1186/s12966-023-01465-7.

## Background

The importance of a healthy diet and active-travel for physical and mental health outcomes is well documented [1, 2], with both sets of behaviours reducing the risk of non-communicable diseases [1–3] and mental illness [4, 5] and active-travel also improving cardiovascular fitness [6, 7]. Focusing on both diet and active travel policies also aligns with the most recent Net Zero agenda to achieve emissions reductions [8, 9]. Implementing effective population level policies, i.e., single voluntary or mandatory interventions, is crucial to achieving sustainable behaviour change in diet and active-travel and improving population health [10–14]. Various diet and active-travel policies have been implemented, which vary in their level of agency (conscious human individual action) or personal resources needed by individuals to benefit from them as intended [15]. Policies making fewer demands on agency, such as change in infrastructure or taxing unhealthy foods and drinks, require less conscious, individual action and have been proposed to be more effective and equitable [16–18]. These policies have been relatively neglected compared to higher-agency policies that require more conscious individual action and aim to educate and provide information, such as educational campaigns and front-of-package (FoP) nutrition labels [16–20]; although they can be complementary and higher-agency policies can contribute to increase acceptance of lower-agency ones [20].

A key issue in implementing and sustaining effective policies, is public and policymaker acceptability, i.e., how members of the public and policymakers think and react to a policy [21]. These are important factors in the decision-making process about which policies might be implemented and can contribute to the effectiveness and sustainability of policies [22, 23]. For example, acceptance of the importance of seatbelts increased, following seat belt legislation, which was associated with increased seat-belt use [24]. On the other hand, policies’ effectiveness may be reduced due to consumers seeking alternative sources to consume affected products, e.g., foods high in saturated fat via black markets or cross-border shopping [25]. Equally, there is evidence showing that policies were repealed after their perceived public and political opposition [26]. In cases of emergency, however, as seen during Covid-19, policy acceptability can be overlooked, which can result in distrust of the government [27], and contribute to reduced compliance with specific measures, e.g., Covid-19 vaccination [28]. Therefore, understanding members’ of the public and policymakers’ views on what matters for the support for diet and active-travel policies may help us gain a greater insight into arguments about acceptability of and opposition to the policies and use this information to effectively communicate policies to maximise their adoption and sustainability [23].

Existing research has shown that the public are more supportive of higher-agency policies, as these are generally considered to maintain more freedom of choice compared to lower-agency policies [29–33]. Public support is also higher for policies perceived to be more effective [34, 35] ) or for policies that have already been implemented [29, 36]. Engagement with the targeted behaviour and embracing pro-health norms are also predictors of acceptability [29, 37]. For example, non-smokers are more supportive of some tobacco control policies, e.g., removing tobacco products from view at the point of sale, compared to smokers [38]. The role of demographic characteristics, such as ethnicity and socioeconomic status, in relation to public support for dietary and active-travel policies is inconsistent; however, women and older people may be more supportive of dietary policies [29–33, 37]. The way policies are framed, e.g. how they are portrayed in the media [39] or whether they are viewed as targeting young people may also influence public support [31].

Policymakers, i.e., people involved in policy decision making and those who devise policy, play an important role in recommending and implementing dietary and active-travel policies. Similar to members of the public, policymakers tend to be more supportive of policies perceived to be effective, [40–42], as well as policies which are feasible, address ‘root causes’ of behaviours, incentivise, rather than penalise behaviour and policies that target people from deprived communities [40, 42].

Existing studies on policy support have primarily used quantitative methods to explore public support for dietary, and to a lesser extent active-travel policies [30, 32, 33, 36, 37, 43, 44]. Qualitative studies have mainly focused on specific policies or levels of agency required to engage with the policy, e.g., taxes [45] or financial incentives [40], specific settings, e.g., schools [46] or supermarkets [47], specific outcomes, e.g., low-calorie foods [48] or less-healthy foods [47], and have mainly been conducted outside the UK [41, 42, 45, 49, 50]. Understanding what shapes public and policymaker acceptability across a broad range of diet and active-travel policies in England is warranted to inform the decision-making process in England and contribute to the effectiveness and sustainability of these policies. The aim of this study was to use a qualitative approach to understand members’ of the public and policymakers’ views on acceptability of various dietary and active-travel policies, explore factors influencing acceptability and how to increase acceptability for these policies.

## Methods

### Participants

#### Members of the public

We sampled adults 18 + years old living in England via an independent market-research agency (https://www.qaresearch.co.uk/). We considered members of the public as any individual of the general population acting in a private capacity. We used purposive sampling to recruit a demographically diverse sample of people across England based on the following characteristics: sex, age, ethnicity (White, Black, Asian or Asian British, Other) and socioeconomic status (SES). We aimed for the sample to be evenly split to the extent it was possible across the different characteristics. SES was assessed via the occupation of the Chief Income Earner according to the social grade classification system (A, B, C1, C2, D, E), to ensure consistency with previous studies, since occupation is typically used as an indicator of SES in the UK [51]. Although there is no consistent guidance to determine sample size in qualitative research, we based our decision on gaining sufficient information power for our research questions, given the breadth of the aim, sample specificity (according to aforementioned characteristics) and rigour of analysis [52]. The market–research agency used a recruitment script to contact members of the public previously enrolled within their research panel and obtain a sample based on the above demographic quotas. The recruitment script was developed by the research team and aimed to provide details about the study, gauge interest for participation, and screen potential participants to obtain our intended sample. An information sheet and a consent form were sent to those expressing interest in participating. Signed consent forms were returned to the research team by 20 members of the public who participated in the study and who received £40 as reimbursement for their time.

#### Policymakers

Policymakers were recruited via existing contacts within our research collaborations and ‘snowball’ sampling with the aim of including individuals working at different levels and roles in local and national public health (e.g., strategy, development, implementation, evaluation and political decision making). We identified policymakers through consultation with members of the research team and by examining the structure of key organisations (e.g., Office for Health Improvement and Disparities). An email was sent to potential participants stating the aim of the study, enclosing an information and consent form. Follow-up phone calls facilitated recruitment and allowed participants to ask questions about the study. We approached 65 people, 11 of whom declined, 6 of these suggested somebody else and 34 did not respond. As such, 20 policymakers returned a completed consent form and took part in an interview. Policymakers were offered a £40 voucher as reimbursement for their time.

### Data collection

**Fig. 1:**
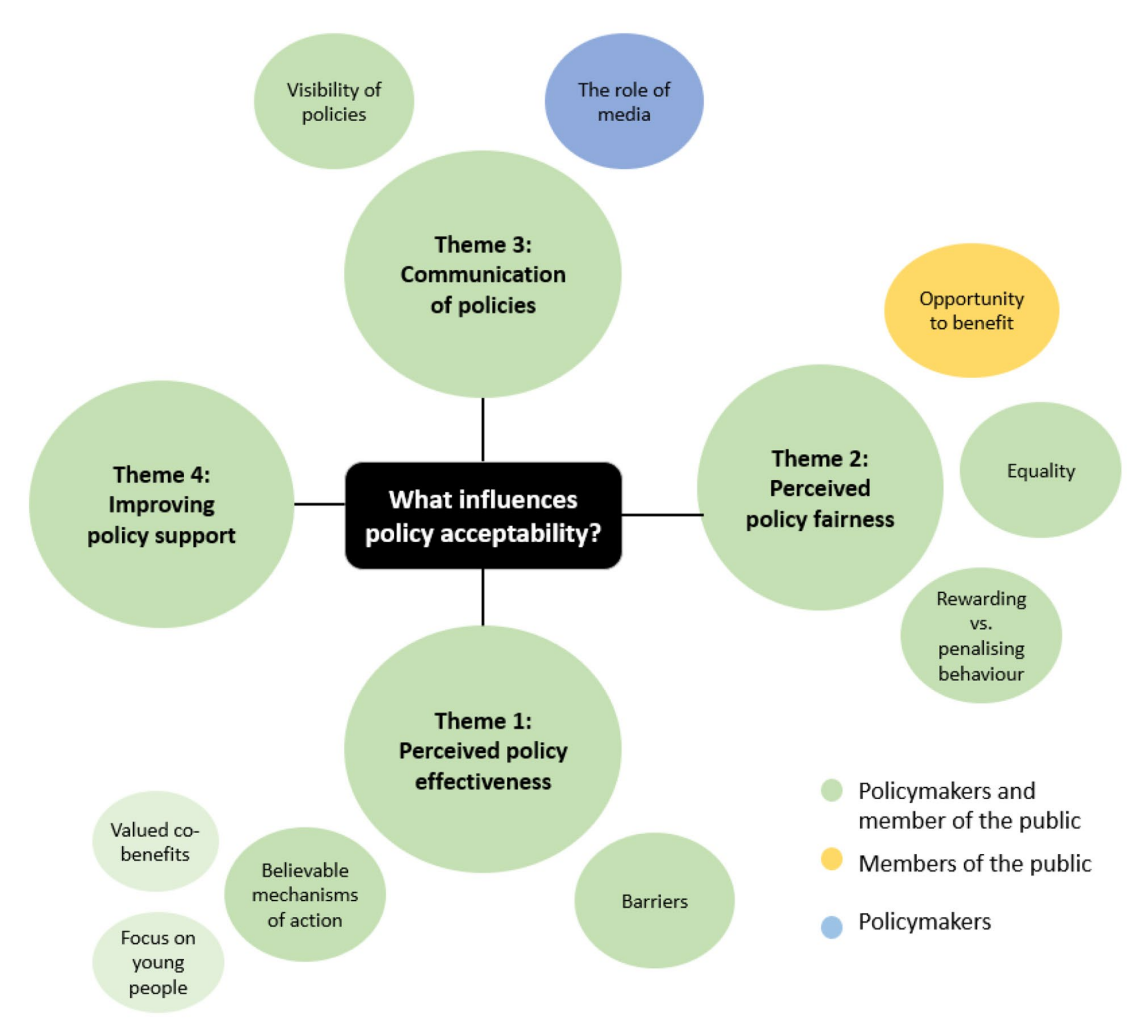
Themes around factors influencing acceptability of dietary and active-travel policies, by members of the public and policymakers

Semi-structured interviews were conducted with all participants with the policymaker interviews conducted later than the public interviews. This allowed for an initial analysis of the public interviews, i.e., familiarising with transcripts, coding and development of themes to inform the interviews with the policymakers. Semi-structured interviews were chosen as they allow focus on key topics, while ensuring that participants can express their views in their own words [53]. We asked participants for their views on acceptability of a wide range of population-level dietary and active-travel policies, some of which exist nationally in England, e.g., Soft Drinks Industry Levy (SDIL), some exist locally in England, e.g., congestion charges and some are not yet implemented in England, e.g., plain packaging of convenience food. This allowed us to capture views on already existing policies, but also new policies with which people would be less familiar, since the stage of policy implementation can influence policy acceptability [29, 36]. These included policies that varied in their scope and focus, i.e., economic, town and city planning, guiding choice, and inform and educate policies **(**Fig. 1, **Supplementary material)**. Examined policies were identified from the literature and have been indicated as effective [14, 19, 41, 54], as well as after consultation with the research team. We developed two interview topic guides (members of the public and policymakers) in consultation with members of the research team (Table 1, **Supplementary material**). Both topic guides asked similar questions but the one for policymakers additionally explored certain issues that members of the public had highlighted, e.g., suggestions members of the public gave to improve policy public support. Questions focused on understanding participants’ views on the acceptability of the various policies; factors affecting acceptability; whether views on acceptability were different depending on how policies affect various population subgroups, e.g., children vs. adults, high vs. low-income groups; and how to increase public support for these policies. Before the interviews, participants were sent a visual aid slide showing the various policies (Fig. 1, **Supplementary material**) and were asked to have this available during the interview.

**Table 1:** Characteristics of members of the public

ID	Sex	Age (years)	Ethnicity	SES (via occupation)
P1	Male	74	White	Intermediate occupations
P2	Male	67	White	Intermediate occupations
P3	Male	25	Black	Routine and manual occupations
P4	Female	49	Black	Higher managerial, administrative and professional occupations
P5	Female	38	White	Intermediate occupations
P6	Male	21	White	Intermediate occupations
P7	Female	44	White	Routine and manual occupations
P8	Male	47	White	Intermediate occupations
P9	Female	70	White	Intermediate occupations
P10	Male	50	White	Higher managerial, administrative and professional occupations
P11	Male	65	White	Higher managerial, administrative and professional occupations
P12	Male	49	White	Routine and manual occupations
P13	Female	72	White	Routine and manual occupations
P14	Female	45	White	Intermediate occupations
P15	Female	36	White	Intermediate occupations
P16	Female	36	Black	Intermediate occupations
P17	Female	69	White	Higher managerial, administrative and professional occupations
P18	Male	58	Asian Indian	Higher managerial, administrative and professional occupations
P19	Male	49	White	Routine and manual occupations
P20	Female	33	Mixed	Intermediate occupations

SES – Socioeconomic status

SES was assessed via the occupation of the Chief Income Earner according to the social grade classification system ((A, B, C1, C2, D, E))

All interviews were conducted by one researcher (KW), who is a British female senior researcher with expertise in physical activity and qualitative methods. Interviews with members of the public took place in May 2021 and interviews with policymakers took place between October 2021-and January 2022. All interviews were conducted via phone or video-conferencing (Zoom). This proved to be a viable tool for collecting qualitative data, offering a convenient experience for both participants and researchers [55]. Interviews were digitally recorded and lasted 40–99 min (Median = 63, IQR (51,80)).

### Data analysis

Interviews were transcribed verbatim by an independent transcription company and anonymised by two researchers (KW, SVP). Transcripts were analysed using reflexive thematic analysis [56, 57]. Transcripts were read to familiarise researchers with the data, after which transcripts were re-read and initial codes were identified inductively [based on meaning of the data [58]] and deductively [based on pre-existing concepts from the literature [29, 30, 32]. Triangulation was employed, where, three transcripts from each participant group were independently read by three researchers (ZT, KW, SVP) and an initial coding framework was created and discussed with other members of the team (ZT, KW, SVP, RJ). The coding framework was then applied to the remaining transcripts by two members of the team (KW, SVP), allowing room for researchers to identify new codes based on the meanings discussed. The research team met regularly to ensure accuracy and consistency, where a few discrepancies were resolved by consensus. Similar codes were then grouped into broader themes with the use of a thematic map, which provided flexibility to move codes around and consider relationships within and between themes. Themes identified were then reviewed to ensure that they were coherent and distinct from one another. Thematic maps of the data were produced to make sense of the relationships between themes (Fig. 2 in the **Supplementary material**). Coding of data was facilitated by NVivo; QSR International Pty Ltd. (version 1.6.1). To reflect on the study’s conceptual rigour, we used the definition of pragmatic saturation by providing rich information on the rationale for our research questions, our process of data collection and analysis, positioning our study alongside previous literature and assuming that the unit of analysis is the concept rather than the participant [59]. This study was approved by the School for Policy Studies ethics and research committee at the University of Bristol (SPSREC/20–21/156). A completed COREQ checklist is presented in the **Supplementary material**.

## Results

Characteristics of members of the public and policymakers are presented in Tables 1 and 2 respectively. We identified four themes (Perceived policy effectiveness, Perceived policy fairness, Communication of policies and Improving public support) (Fig. 1). There were several overlapping thoughts and reactions to policies between members of the public and policymakers, however two sub-themes (the role of media and opportunity to benefit) were only discussed by policymakers and members of the public respectively. We did not observe any differences in patterns of acceptability across the different policy groups, participants were mostly focusing on the acceptability of individual policies presented. Illustrative quotes for each theme and sub-theme, with information on ‘sex/age/socioeconomic status/ethnicity’, are presented in Table 2 of the **Supplementary material**.

**Table 2:** Characteristics of policymakers

	Role	Organisation	Sex	ID
London	Subject Leader	National Agency	Female	PM1
Northeast	Councillor	Local authority	Male	PM2
	Assistant Director of Public Health	Local authority	Female	PM5
	Director of Public Health	Local authority	Female	PM6
	Advanced Public Health Practitioner	Local authority	Male	PM9
	Councillor	Local authority	Female	PM13
	Councillor	Local authority	Male	PM14
Southwest	Public Health Specialist	Local authority	Female	PM3
	Specialist Public Health Manager	Local authority	Female	PM4
	Consultant in Public Health	Local authority	Female	PM7
	Public Health Specialist	Local authority	Male	PM8
	Public Health Specialist	National Agency	Female	PM10
	Senior Public Health Practitioner	Local authority	Male	PM11
	Built Environment Lead	Local authority	Female	PM12
	Advanced Public Health Practitioner	Local authority	Male	PM15
	Health Improvement Coach	Local authority	Female	PM16
	Councillor and Cabinet Member	Local authority	Female	PM17
	Health improvement Coach	Local authority	Female	PM18
	Head of Commissioning (Health Improvement)	Local authority	Female	PM19
	Spatial Planning and Public Health Officer	Local authority	Male	PM20

## Theme 1: perceived policy effectiveness

Both members of the public and policymakers favoured policies, which they perceived to be effective. For members of the public perceived effectiveness was based on their personal experience, e.g., they have ‘witnessed’ no reduction in sales of cigarettes after plain packaging, hence they did not support plain packaging of convenience food, whereas for policymakers according to their understanding of ‘research evidence’ and perceived uptake of policies. Members of the public mainly believed that tax breaks and educational policies like Change for Life (C4L) campaign were effective but additionally highlighted perceived ineffective policies, such as plain packaging of convenience food and building cycle lanes, due to their perceived limited usage.

Policymakers believed that there is stronger evidence for the effectiveness of some lower agency policies such as the SDIL and congestion charging, i.e., paying a fee for driving in central parts of a city, in contrast to inform and educate policies, such as educational campaigns, whose effectiveness was perceived to be limited because the emphasis of changing behaviour is placed on the individual. For guiding choice policies, such as plain packaging of convenience food and removing sweets from checkouts, views around perceived effectiveness were mixed, with some policymakers believing that there is strong evidence that they work, whereas others were more cautious, highlighting the need to build a stronger evidence base.

### Believable mechanisms of action

Members of the public supported policies when they could describe a hypothesised believable mechanism of action in changing behaviour, i.e., how a policy works to achieve behaviour change. Mechanisms that people understood and believed were: improving finances (e.g., subsidies improve access to ‘expensive’ fruit and vegetables, tax breaks help save money); making the physical environment safer to cycle (building cycle lanes); reducing temptation and pestering of parents (removing sweets from supermarket checkouts and plain packaging of convenience foods); reducing accessibility to takeaways (preventing new takeaways near schools); making information visually appealing and easy to understand (FoP nutrition labels) and raising awareness about healthy eating and physical activity choices (C4L campaign). A few members of the public, as well as one policymaker, believed that policies like preventing new takeaways near schools, building cycles lanes and educating children also work via changing the norms of certain behaviours, e.g., eating unhealthily with friends after school.

In contrast, members of the public perceived the reduction in portion sizes of convenience food as an ineffective policy, due to an unconvincing mechanism of action, i.e., they believed that current portion sizes were not large enough and hence a further reduction in portion size would lead people to buy more, not less, food.

Introducing maximum working hours was supported by some members of the public and policymakers as a ‘visionary’ policy and was believed to operate via promoting ‘work-life balance’ and better ‘living conditions’, which would then have a positive impact on dietary and physical activity behaviours. However, some members of the public and policymakers expressed concerns that the additional time gained would not be used for the preparation of healthier meals and exercise, hence the policy would not work as intended.

Finally, the mechanism of action for the SDIL was perceived to be different between policymakers and members of the public, i.e., members of the public highlighted that the SDIL works via increasing products’ cost, whereas policymakers discussed how it works via product reformulation.

#### Valued co-benefits

Members of the public also discussed how policies work via achieving co-benefits that they valued. These were mostly in relation to active travel policies, like congestion charging and building cycle lanes, which were particularly supported because of their perceived positive impact on the environment, e.g., improving air quality and congestion. However, few members of the public noted how cycle lanes had the opposite effect of increasing congestion due to the narrower lanes used for cars. One member of the public also highlighted how the SDIL was useful in increasing revenue for the government.

Policymakers similarly supported building cycle lanes and congestion charging because of their positive impact on the environment and congestion and further highlighted how some policies work via making people ‘environmentally aware’. For example, they supported policies that reduce waste and packaging and policies that promote community gardens and growing spaces, because they foster a strong relationship with nature. They believed that these ‘green space’ policies, alongside other policies aligned with climate change contribute to a more aesthetically pleasing and ‘greener’ physical environment, which members of the public would value and subsequently support. One policymaker also mentioned how educational policies like C4L can also have an additional benefit to mental health because of eating healthier and being physically active.

#### Focus on young people

There were inconsistent findings about whether policies should be targeted at young people to be more effective and better supported. Some members of the public said that they would better support policies if they were targeted at young people, for example inform and educate policies, building cycle lanes and removing sweets from checkouts. They believed that targeting younger age groups would work better by establishing healthy habits early, which would positively impact on adult health. However, some members of the public also expressed the view that policies should be targeted ‘across the board’, since optimal diet and physical activity are equally important for both young people and adults.

Policymakers highlighted that there is now a focus on early years and tackling childhood obesity, as they also believed that setting healthy habits from a young age will contribute to better health in later life. However, they also acknowledged that policies should also target adults to be able to holistically tackle public health issues like obesity.

### Barriers

Both members of the public and policymakers discussed similar barriers perceived to hinder policy effectiveness. The effectiveness of active-travel policies was believed to mainly be hindered by inadequate infrastructure, safety concerns and bad weather. Barriers to dietary policies included limited cooking skills, ‘unhealthy’ food marketing and the variation in FoP label designs making access to information more challenging. The effectiveness of all policies was perceived to be limited by financial barriers (e.g., high cost of bikes and ‘healthy’ food), lack of education and existing mentality and culture relating to poor food and physical activity choices.

## Theme 2: perceived policy fairness

Perceived policy fairness was important for policy acceptability and was discussed in relation to three dimensions: opportunity to benefit, equality and rewarding vs. penalising behaviour. There was no consistent evidence on which policies were perceived as ‘fair’ overall, as often they only satisfied certain elements of perceived fairness, e.g., inform and educate policies like C4L were believed to provide opportunities for everyone to benefit but were also believed to be ‘unequal’ by only reaching those with a high education background.

### Opportunity to benefit

Members of the public believed that ‘fair’ policies were the ones that provide everyone with an equal opportunity to engage and benefit from the policy. Such policies were the ones that made resources available to all and were subsequently considered to increase everyone’s opportunities to benefit. For example, building cycle lanes and providing information via C4L and FoP nutritional labels were believed to be free and widely available resources that everybody could use and benefit from.

### Equality

Members of the public referred to policies as ‘fair’ if they applied to all people and did not exclude anyone based on different needs. A few members of the public referred to building cycle lanes and the inform and educate policies, e.g., C4L and FoP labels, as being applicable to everyone regardless of their demographic or behavioural background, since they believed that everyone could engage with these policies and benefit. In contrast, some members of the public perceived preventing new takeaways near schools as ‘unfair’ towards independent food businesses, since these would be impacted more severely compared to large fast-food chains opening takeaways.

Most members of the public, as well as policymakers, referred to policies which were perceived to be ‘unfair’, because they did not consider everyone’s needs or were believed to only be applicable to certain population subgroups. For example, congestion charging was perceived to disadvantage people living or working in the city centre and people with low income who cannot afford to pay charges or use public transport, which was perceived to be expensive. Building cycle lanes and the Cycle2Work scheme were believed to not consider people with a long commute to work, older people and people unable to cycle. Introducing maximum working hours was believed to not consider people whose jobs required long shifts and those with hourly paid contracts. C4L and FoP nutritional labels were believed to not consider people with lower education backgrounds and C4L specifically was believed to mainly target British white families.

Policymakers also discussed inform and educate policies, as perceived ‘unequal’ policies, and believed that these policies were targeting people who are already better equipped to make healthier choices and have the cognitive resources to process information provided.

### Rewarding vs. penalising behaviour

Both members of the public and policymakers expressed better support for policies that encouraged and financially incentivised healthy behaviours, e.g., tax breaks and subsidies for fruit and vegetables, as opposed to policies that only ‘punished’ unhealthy behaviours, e.g., congestion charging and a reduction in portion size of convenience foods. Policies that only penalised unhealthy behaviours, e.g., reducing portion size of convenience foods were seen as ‘unfair’, because they were not equally considering and rewarding people engaging in healthy behaviours. Both groups of participants therefore argued that if ‘punitive’ policies need to be implemented, these should be alongside policies that ‘give something back’, i.e., reducing portion size of convenience foods should be implemented alongside a reduction in the cost of non-convenience food.

## Theme 3: communication of policies

How policies are communicated and advertised was believed to be important for their acceptability. Both members of the public and policymakers highlighted the importance of ‘visible’ policies that are effectively communicated to become known to members of the public. Additionally, policymakers discussed the role of media and how policy-related information is portrayed that may influence policy acceptability.

### Visibility of policies

Both members of the public and policymakers discussed how certain policies are made more ‘visible’ compared to others and therefore may be better supported. Members of the public mainly talked about C4L as a poorly advertised and communicated policy, which resulted in people not being aware of its purpose. A few people who were familiar with C4L mentioned how they needed to be prompted to search and engage with it, e.g., by health professionals.

Policymakers described how certain policies, especially infrastructure related ones like building cycle lanes, are more ‘visible’ and therefore better supported, compared to ‘invisible’ policies like the economic ones. They additionally expressed concern about the effectiveness of current policy communication strategies, as they often felt that the even perceived ‘positive’ policies, such as offering governmental grants for energy efficiency measures, were not being relayed to members of the public, which resulted in the policies’ low uptake.

### The role of media

The role of media was only discussed by policymakers. Policymakers emphasised how policy acceptability can be influenced by the way media choose to portray each policy and highlighted that media was often responsible for incorrect messages. For example SDIL and preventing new takeaways near schools had received negative publicity because of their potential adverse impact on disadvantaged communities or inconsistent messages, i.e., the SDIL will work via reformulation or by increasing the price of sugar-sweetened beverages.

## Theme 4: improving policy support

Members of the public and policymakers made several recommendations on how to improve policy support, which were aligned with each of the three aforementioned themes of ‘perceived policy effectiveness’, ‘perceived policy fairness’ and ‘communication of policies’. Specific recommendations are mapped onto each of the previous themes in Table 3.

**Table 3:** Recommendations to increase public acceptability of policies by members of the public and policymakers mapped onto each theme

Theme/ Sub-theme	Members of the public	Policymakers
**Theme 1: Perceived policy effectiveness**		
Believable mechanisms of action	None.	Explain mechanism by which policy intends to modify behaviour (mixed views on this suggestion, as some PMs worried that the mechanism of action would reveal unconscious thought processes, e.g., removing sweets from checkouts).
Barriers	Make public transport accessible/convenient and affordable (e.g., financial schemes to buy and fix bikes).	Make public transport accessible/convenient and affordable (e.g., financial schemes to buy and fix bikes).
	Invest in cycling infrastructure (segregated and covered cycling paths, showers, bike racks).	Invest in cycling infrastructure (segregated and covered cycling paths, showers, bike racks).
	Invest in cycling lessons.	Provide incentives for active-travel (e.g., financial/reduced working hours).
	Provide incentives for active-travel (e.g., financial/reduced working hours).	Ensure safety by increasing policing in public spaces, e.g., parks.
	Ensure safety by increasing policing in public spaces, e.g., parks.	Ban or regulate ‘unhealthy’ food availability and marketing.
	Ban or regulate ‘unhealthy’ food availability and marketing.	Extend taxes to other ‘unhealthy foods’.
	Increase proposed distance for takeaways around schools.	Food reformulation.
	Provide financial aid for healthy foods.	Provide financial aid for healthy foods.
	Invest in cooking and meal planning lessons.	Invest in cooking and meal planning lessons.
	Provide health education.	Provide health education.
Valued co-benefits	Better communicate benefits of policies on current concerns (e.g., mental health).	Better communicate benefits of policies on current concerns (e.g., mental health).
**Theme 2: Perceived policy fairness**		
Opportunity to benefit	None.	None.
Equality	Design policies that consider individual differences (e.g., employment status, job type, socioeconomic status, age, ethnicity).	Design policies that consider individual differences (e.g., employment status, job type, socioeconomic status, age, ethnicity).
		Implement and prioritise multisectoral policies, e.g., across public transport, health, education.
		Design policies with exemptions for certain populations.
		Tackle inequality (food insecurity, poverty and low living wages).
Rewarding vs. penalising behaviour	Introduce maximum working hours with no reduction in salary.	Reduce portion sizes while reducing prices.
	Reduce portion sizes while reducing prices.	Promote and provide awards for healthy takeaways.
	Combine policies with penalties with policies that provide rewards.	
**Theme 3: Communication of policies**		
Visibility	Communicate policy successes.	Communicate policy successes.
	Use recommended channels to transmit message: word of mouth, social media, health champions (e.g., Marcus Rashford and Jamie Oliver).	Involve members of the community in policy communication to make them more visible and reach more people.
The role of media	None.	Messages should be non-judgemental, consistent, accurate and mention short-term effects .

Both the members of the public and policymakers argued for the implementation of multiple policies to address various sides of dietary and active-travel behaviours. It was believed that individual policies cannot solve the problem and an integrated strategy of policies was needed to improve these behaviours. Both groups of participants also highlighted how policies should address barriers they had previously identified, e.g., make nutritional information more accessible and easier to understand, as well as improve infrastructure and public transport.

Both groups of participants argued for inclusive policies, which consider everyone’s individual needs. Examples of inclusive policies would be policies that equally target people regardless of their demographic or health background, for example their employment status or having a disability. They also argued for policies that do not only ‘punish’ unhealthy behaviours but equally financially reward people who engage in healthy behaviours.

Changes to the way policies are communicated were believed to improve their acceptability, by raising awareness of the policies among members of the public and by clearly outlining their purpose and impact on behaviour via clear and consistent messages. Members of the public mainly recommended using appropriate channels (e.g., social media and health champions) to raise awareness about policies, and policymakers focused on involving the community in policy communication and presenting consistent messages from the media.

## Discussion

We used a qualitative approach to explore public acceptability of a broad range of dietary and active-travel population policies by members of the public and policymakers in England. Dietary and active-travel policies were better supported when they were perceived as effective, i.e., they had believable mechanisms of action, valued co-benefits and addressed multiple barriers of the behaviour; fair, i.e., they provided everyone with an opportunity to benefit (mentioned only by the public), equally considered everyone’s needs and rewarded ‘healthy’ behaviours rather than penalising ‘unhealthy’ behaviours’; and were clearly communicated, i.e., they were visible and had received consistent and correct messages in the media (mentioned only by policymakers). Evidence on framing policies as targeting young people was inconsistent, as some participants argued for policies being effective by targeting people early and others argued that effective policies need to be inclusive and include both young people and adults. Participants provided recommendations on how to increase public support for the examined policies, which could be used to develop effective communication strategies to maximise policies’ effectiveness and sustainability. We did not observe any patterns of acceptability across the different policy groups examined, e.g., guiding choice vs. inform and educate policies, showing that people in this study tended to focus on the acceptability of individual policies. This might suggest that acceptability should be assessed for each policy separately, although further research to assess acceptability between groups of policies with similar characteristics is needed.

Our findings align with previous international studies [42, 45, 49, 50] among policymakers, that have acknowledged the need for an integrated strategy to address multifaceted behaviours, i.e., obesity, diet and active travel, rather than individual policies. Our findings also agree with previous research that has shown perceived effectiveness, fairness and media representation of policies, as important factors influencing public and policymaker acceptability [29–33, 35–37, 39], as well as the importance of framing policies alongside policy co-benefits [60]. Our research enhance these previous findings, by adding further insight on what aspects members of the public and policymakers perceive as important when they refer to the effectiveness, fairness and communication of policies.

Perceived effectiveness was the most frequently mentioned theme linked to acceptability, with both members of the public and policymakers supporting policies perceived as effective. Communicating evidence of policy effectiveness is suggested to increase public support [29, 34, 35, 43, 48] by changing underlying beliefs and attitudes about policies [29, 34]. Members of the public perceived policies as effective when these included a believable mechanism of action. However, identified perceived mechanisms of action were not always accurate, e.g., members of the public believed that the SDIL works via increasing beverages’ cost rather than reformulation, although this was not supported by policymakers. This suggests that mechanisms of action need to be primarily believable to the public to support a policy, although they are not always accurate. Hence, future policy strategies should aim to explore mechanisms of action that people understand and believe, as well as rectify incorrect messages, to maximise acceptability.

Policies with valued co-benefits, e.g., tax breaks that positively impact on climate change, were better supported by both groups of participants, which could reflect that policies with multiple co-benefits are seen as more effective. Previous research has shown that communicating multiple benefits of policies on energy-dense foods, meat and alcohol increased their public support [60] and our findings extend this notion to policymakers. This could be because policymakers favour policies that fulfil intersectoral objectives (‘win-win’ strategies), due to the sharing of resources and collaboration to achieve each sector’s goals [61].

Both members of the public and policymakers highlighted several barriers that hindered perceived policy effectiveness, suggesting that diet and active-travel are considered to be multifactorial behaviours, whose perceived effectiveness is related to simultaneously addressing numerous diverse factors and mechanisms of action. Implementing single policies was not favoured by either group of participants, who argued for the implementation of an integrated strategy of a collection of policies to improve diet and active-travel. For example, taxing foods and drinks high in fat, salt or sugar (HFSS), could be implemented alongside regulations restricting HFSS food and drink advertising [48], as well as effective cooking programmes to increase confidence and skills [49].

Perceived policy fairness has been previously suggested as a core value for its acceptability [29, 43, 62], which aligns with our findings. However, we shed additional light into what this term encompasses, by describing three dimensions in relation to perceived policy fairness. Members of the public believed that policies, like building cycle lanes and inform and educate policies, were fair because they made resources available and hence provided everyone with an opportunity to benefit. However, access and use of these resources can be socioeconomically patterned [63, 64] and hence this belief incorrectly equates availability of resources to accessibility. Participants also believed that perceived fair policies should be inclusive and equally consider everyone’s needs according to their demographic and behavioural background, e.g., socioeconomic status and disability. This suggests that policymakers and more importantly members of the public are likely aware of structural issues influencing dietary and active-travel behaviours, which policies should aim to address. Finally, perceived fairness was discussed in relation to policies that financially rewarded ‘healthy’ behaviours rather than only penalising ‘unhealthy’ behaviours. Previous research has shown that both members of the public and policymakers express some, but not overwhelming, support for financial incentives for ‘healthy’ behaviours [40, 65, 66], however our findings did not indicate any opposition to such policies. This could be because financial incentives were not the sole focus of this study, although policies examined included perceived ‘punitive’ approaches, e.g., congestion charging. Our findings add value by suggesting that arguments about financial rewards and penalties are not only related to policies with a clear financial focus, e.g., subsidies or congestion charging, but can extend to other policies, e.g., reduction in portion size of convenience foods.

Policies’ perceived effectiveness and fairness were considered important for acceptability by both members of the public and policymakers. For some policies, there were both positive and negative connotations of their perceived effectiveness and fairness, for example, members of the public perceived building cycle lanes as an ineffective policy due to its perceived limited uptake, but they believed it was a ‘fair’ policy, since they made cycle lanes available for everyone to benefit. This made it difficult for participants to decide on overall support for a policy. Previous research suggests that presenting counter arguments in relation to obesity policies, for example policy narratives that emphasise individual responsibility vs. policy narratives that emphasise wider determinants of obesity, has resulted in higher levels of support [67]. Future studies should therefore examine to what extent presenting information with mixed connotations of policy attributes, e.g., policies that are ineffective but ‘fair’, may influence public support.

Clear communication of policies was perceived to be important for their acceptability, although both policymakers and the public believed that this was currently ineffective. The public especially referred to C4L campaign as a perceived unadvertised policy, whose name was familiar but they were often unaware of its purpose. A recent cross-sectional study of 5,466 adults in the UK [68] showed that 18% of participants indicated awareness of public health campaigns and only 3% mentioned C4L as one such campaign, mainly people from higher education backgrounds. Policymakers focused on the role of media and how it can enhance or thwart policy perceptions and highlighted incorrect and inconsistent messages. Future communication strategies should aim to improve communication of policies by using alternative channels and messages (suggested by members of the public and policymakers respectively), as well as engage the community in policy communication.

### Strengths and limitations

A strength of this study is the use of a qualitative approach to examine both diet and active-travel policies, as well as the inclusion of both members of the public and policymakers to arguments of acceptability and opposition of such policies. Our focus on both diet and active travel offers added value, since our research provides insights on policy acceptability of complementary behaviours that can improve physical [1–3], mental [4, 5] and planetary health [8, 9]. We also considered both dietary and active-travel policies that varied in their level of agency and focus e.g., economic and guiding choice. Furthermore, most other qualitative investigations of acceptability of dietary and active-travel policies were conducted in the USA and Australia, therefore our study of eliciting stakeholders’ views in England provides a novel lens to examine the issue of policy acceptability. Our members of the public sample included males and females from a wide age range, however most participants were from a White and high SES background, which may have limited the views expressed and did not allow us to explore differences across different characteristics. Interviews being conducted via Zoom may have limited our ability to recruit more members of the public from low SES groups and ethnic minorities. It was not possible to obtain members’ of the public reasoning for refusing to participate in the study, which would have helped address these issues in future studies. Our policymaker sample was drawn from the Southwest and North-East of England to account for different policy priorities, however it only included four elected councillors. Having more locally elected councillors might have shed additional light on factors influencing public support from a decision-making perspective. Our sample did not include members from private organisations, e.g., corporations, whose views might have highlighted different issues around public support for dietary and active-travel policies. Although we examined different types of policies based on status (existing vs. not yet implemented in England), scope (economic, town and city planning, guiding choice, inform and educate) and effectiveness, there were other example policies that we did not focus on, e.g., banning adverts online or ‘get off the bus early’ campaign. These might have revealed different themes around acceptability and hence, future studies should aim to explore these findings in relation to other policies. Finally, these are the views of some members of the public and policymakers in England, and therefore the findings may not be generalisable to other settings or populations.

## Conclusions

Our findings highlight that public support can be shaped by perceived policy effectiveness, perceived fairness and the way policies are communicated. Our findings shed additional light into what aspects of perceive effectiveness, fairness and policy communication members of the public and policymakers perceive as important in relation to acceptability and suggest ways on how to increase support for dietary and active-travel policies. They highlight the need for an integrated strategy of inclusive policies, which have believable mechanisms of action, address multiple barriers and valued co-benefits, as well as inclusive policies that equally consider everyone’s needs, reward ‘healthy’ behaviours and are clearly communicated. These findings can inform the policy agenda on aspects to consider in policy implementation, but also help develop effective communication strategies to increase acceptability, effectiveness and sustainability of dietary and active-travel policies.

## Electronic supplementary material

Below is the link to the electronic supplementary material.

Supplementary Material 1

Supplementary Material 2

## Data Availability

The datasets used in the current study are stored in a University of Bristol secure repository and can be shared by the author on request.

## List of abbreviations

AT: Active-Travel
C4L: Change4Life
FoP: Front-of-Pack
PA: Physical Activity
SDIL: Soft Drinks Industry Levy
SES: Socioeconomic Status
