# Supplementary material for: Exploring views of members of the public and policymakers on the acceptability of population level dietary and active-travel policies: a qualitative study
Source: Int J Behav Nutr Phys Act. 2023 May 31;20:64. doi: 10.1186/s12966-023-01465-7 (PMC10230483; doi:10.1186/s12966-023-01465-7)
Supplement: Supplementary file 2 — Supplementary Material 2 [file 12966_2023_1465_MOESM2_ESM.docx]

**‘Exploring views of members of the public and policymakers on the acceptability of population level dietary and active-travel policies: a qualitative study.’**

**Supplementary material**

**Table 1: Interview topic guide for members of the public and policymakers**

| **Members of the public** | | **Policymakers** | |
| --- | --- | --- | --- |
| Ice breaker | | Ice breaker | |
| Economic policies: sugar tax, subsidies for fruit and vegetables, congestion charging, tax breaks for bus/bike commuting, Cycle2Work scheme | Do you support these policies?   - Why/why not? | Economic policies: sugar tax, subsidies for fruit and vegetables, congestion charging, tax breaks for bus/bike commuting, Cycle2Work scheme | Do you support these policies?   - Why/why not? |
|  | Do you think these policies are fair towards all groups of people?   - Why/why not? - If not, can you please give me examples of groups of people who may be disadvantaged by these policies? |  | Do you think these policies are fair towards all groups of people?   - Why/why not? - If not, can you please give me examples of groups of people who may be disadvantaged by these policies? |
|  | If these policies were exclusively focused on children and young people, would you support them more, equally or less?   - Why/why not? |  | Members of the public have suggested that these policies could become more acceptable by, X, Y, Z, – what do you think of these suggestions? |
|  | How would you improve these types of policies to make them more acceptable to the public? | Town and city planning policies: preventing new takeaways within 400m of a school, building cycle lanes on busy roads | Do you support these policies?   - Why/why not? |
| Town and city planning policies: preventing new takeaways within 400m of a school, building cycle lanes on busy roads | Do you support these policies?   - Why/why not? |  | Do you think these policies are fair towards all groups of people?   - Why/why not? - If not, can you please give me examples of groups of people who may be disadvantaged by these policies? |
|  | Do you think these policies are fair towards all groups of people?   - Why/why not? - If not, can you please give me examples of groups of people who may be disadvantaged by these policies? |  | Members of the public have suggested that these policies could become more acceptable by, X, Y, Z, – what do you think of these suggestions? |
|  | If these policies were exclusively focused on children and young people, would you support them more, equally or less?   - Why/why not? | Guiding choice policies: removing sweets from supermarket checkouts, reducing portion sizes of convenience foods, introducing plain packaging for convenience foods, introducing maximum working hours to give more time for people to exercise and prepare food | Do you support these policies?   - Why/why not? |
|  | How would you improve these types of policies to make them more acceptable to the public? |  | Do you think these policies are fair towards all groups of people?   - Why/why not? - If not, can you please give me examples of groups of people who may be disadvantaged by these policies? |
| Guiding choice policies: removing sweets from supermarket checkouts, reducing portion sizes of convenience foods, introducing plain packaging for convenience foods, introducing maximum working hours to give more time for people to exercise and prepare food | Do you support these policies?   - Why/why not? |  | Members of the public have suggested that these policies could become more acceptable by, X, Y, Z, – what do you think of these suggestions? |
|  | Do you think these policies are fair towards all groups of people?   - Why/why not? - If not, can you please give me examples of groups of people who may be disadvantaged by these policies? | Inform and educate policies: Front-of-pack nutrition labelling, the Change4Life campaign | Do you support these policies?   - Why/why not? |
|  | If these policies were exclusively focused on children and young people, would you support them more, equally or less?   - Why/why not? |  | Do you think these policies are fair towards all groups of people?   - Why/why not? - If not, can you please give me examples of groups of people who may be disadvantaged by these policies? |
|  | How would you improve these types of policies to make them more acceptable to the public? | Do you have any questions? | |
| Inform and educate policies: Front-of-pack nutrition labelling, the Change4Life campaign | Do you support these policies?   - Why/why not? |  | |
|  | Do you think these policies are fair towards all groups of people?   - Why/why not? - If not, can you please give me examples of groups of people who may be disadvantaged by these policies? |  |  |
|  | If these policies were exclusively focused on children and young people, would you support them more, equally or less?   - Why/why not? |  |  |
| Do you have any questions? | |  |  |

**
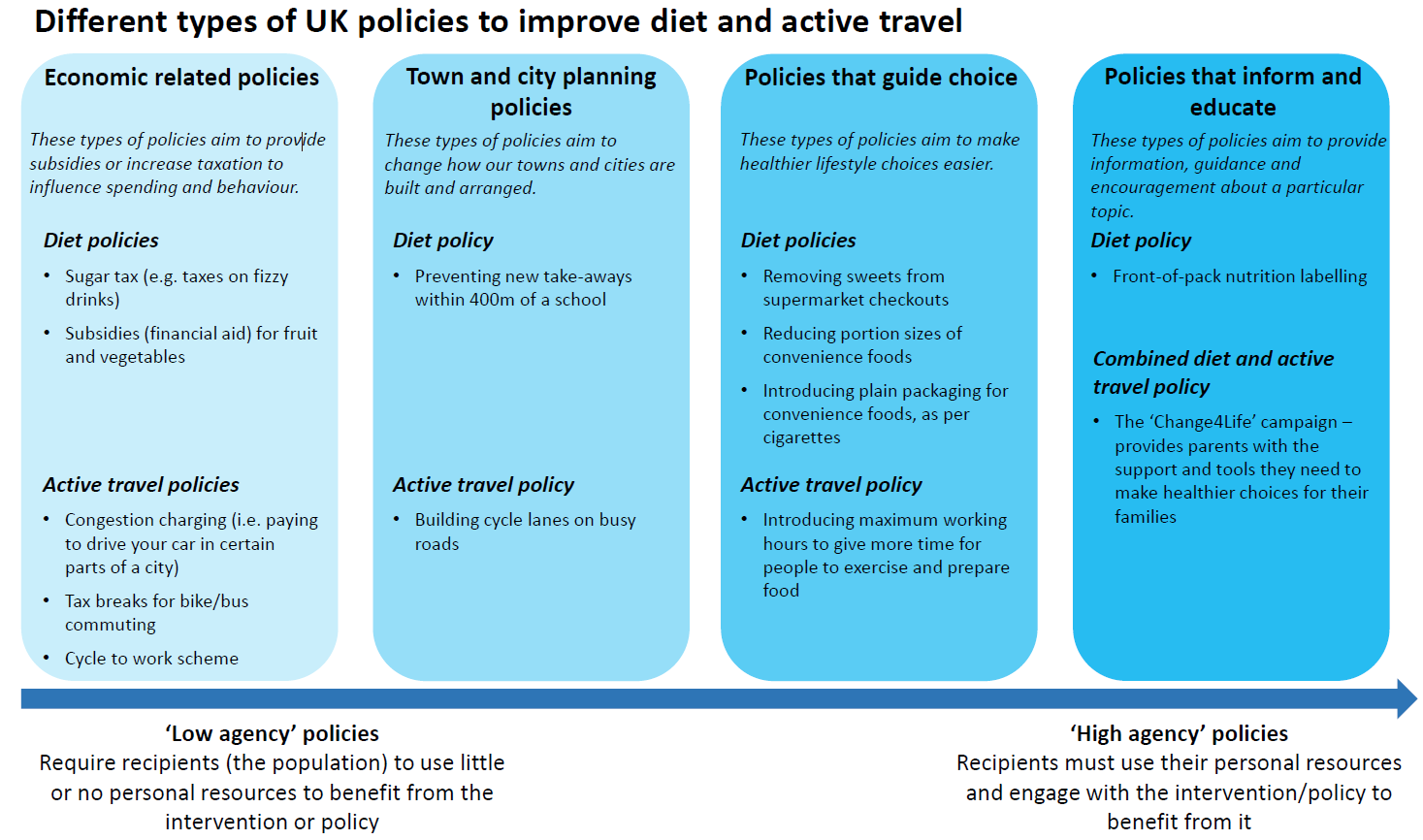
Figure 1:** **Policies examined – visual aid presenting the various policies used during interviews to help participants view the policies**

**Figure 2: Early thematic map to make sense of developed themes.**


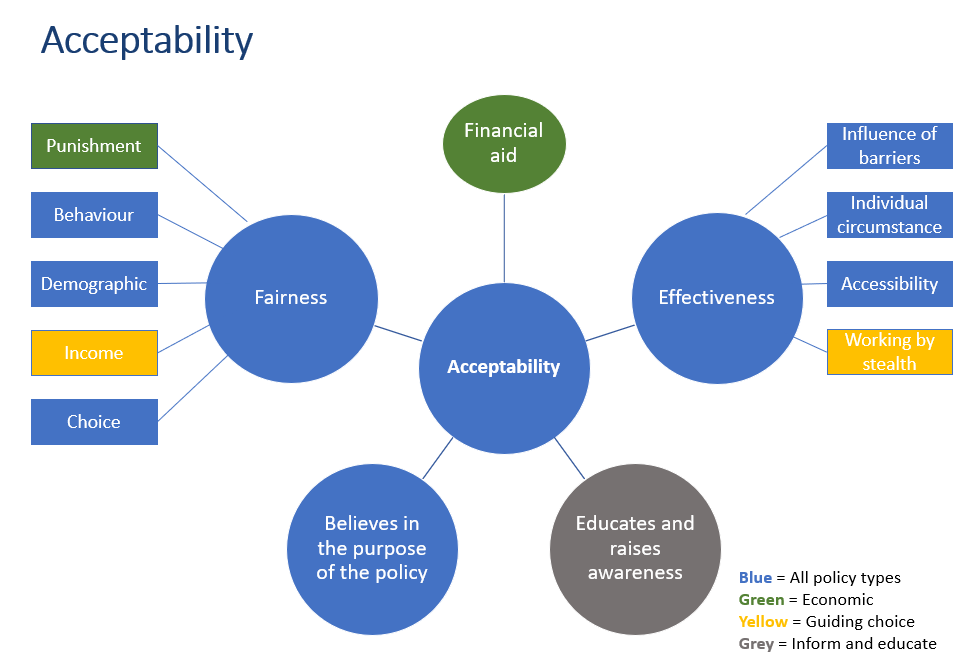


**Table 2: Illustrative quotes for each theme and sub-theme by members of the public and policymakers.**

|  | **Members of the public** | **Policymakers** |
| --- | --- | --- |
| **Theme 1: Perceived policy effectiveness** | | |
|  | I don't think it would make much difference [packaging of convenience food] I used to work in a shop and the plain packaging on cigarettes didn't make any difference [P9, F/60-79/Intermediate/White] | I [am] largely in favour [SDIL]. I think we’ve demonstrated through Public Health evidence that taxation-based schemes, certainly in terms of sugar tax, have worked at a population level in other countries [PM5] |
|  | I mean, I do pass them [cycle lanes] and I never see any bicycles on them [P13, F/60-79/Low/White] | In terms of congestion charging, I think there's a decent amount of evidence we’ve got now that there are generally positive effects on things we'd like to do in terms of active travel and managing congestion. [PM20] |
|  | The Cycle2Work scheme actually helped that because bikes are so expensive. […] That really helped him [my dad] get into cycling. Now he actually will cycle to work one day […] I’ve seen that work myself, in my own household so… [P6, F/20-39/Intermediate/White] | I think that's where some of [guide choice policies], we're showing what we think make sense and what we know that there's some limited evidence, but there's not necessarily a big chunk of evidence to show that actually, we will get the outcomes that we expect. [PM3] |
|  |  | But especially removing sweets from supermarket checkouts, because it’s that immediate making the healthier choice the easier one. And I genuinely, in my own opinion, believe it works. Because if it’s not in line of sight, especially in places like Aldi and Lidl, who have really adopted that strategy. [PM18] |
| Believable mechanisms of action | That's good I think, if that's something that's happening [subsidies] I think that's really good because I feel like so many people don't buy fresh fruit and vegetables because they're so expensive [P16, F/20-39/Intermediate/Black] | I think people feel that eating healthier is more expensive than eating more poorly. […] That's a separate topic, but I think policies where fruit and vegetables are subsidised, or like the Healthy Start vouchers, I think, do encourage people, in my experience, to eat more vegetables. [PM16] |
|  | And for the people that cycle it would make a difference because they can be safer knowing they've got a specific, dedicated lane for them [P16, F/20-39/Intermediate/Black] | People don't know it. It comes as a complete surprise to them when you start telling them this stuff and what is in the food that they are eating. They don't know it, so I think we really need to educate people if we want them to make different choices. [PM16] |
|  | I’d be for that [SDIL] […]. So, actually by introducing some tax, and if that does then have the effect of raising the cost, which then reduces people consuming it, then yes, I think that’s a sensible approach [P8, M/40-59/Intermediate/White] | If they're just not by the checkouts, it means that kids are less likely to pester their parents while they're waiting in a queue. [PM19] |
|  | Yes. As I said, the Change4Life campaign was very good, it gave the information. It pricked my conscience and I’m sure that it will have done the same to a lot of people and made them think of exercising. [P2, M/60-79/Intermediate/White] | And I think they make a difference. We know that cycle lanes help people commute in an easier manner, and they feel safer doing so. [PM7] |
|  | I think that’s a good start though, that people can see sugar content and things. It’s got to be a visual thing, for them to be able to- so that they understand what it is that they're eating, how much sugar is in things. [P14, F/40-59/Intermediate/White] | I mean, actually for a start, I kind of know it because the revenue hasn’t been so great, has it? That’s because it’s forcing manufacturers to reformulate in the main [PM9] |
|  | It's too easy for you to pick up, and at the front, some of them, they've got bags of crisps and stuff like that. Yes, remove, definitely [P19, M/40-59/Low/White] | plain packaging. I think this is a big one for children, because they are often visually-driven. So, I think that would help parents, because kids often see these bright colours and pictures and will pick it up and ask their parents to buy it. Whereas if it was plain, I don't think it would get as much attention. [PM18] |
|  | Anything else that you buy is convenience, microwaved food, I would say you need to double up if you were going to actually buy to feed two pretty normal eating adults. I think reducing them is not necessarily- I don’t know. I don’t think that would work. [P17, F/60-79/High/White] | I think things like the [maximum working hours] policy there, that is lovely, feels very visionary, feels like it would be really hard to get buy-in on something like that, but would be a lovely thing to do. And if you can have a couple of businesses locally showing best practice, that would be great. [PM3] |
|  | Because it’s not tempting, is it? It’s not appetising, I guess. I’m sure if all the chocolates and sweets on the front of the checkouts were all in plain packaging, my children probably wouldn’t even cause such a fuss [P20, F/20-39/Intermediate/Mixed] | Any changes that have an improvement on people's living conditions and experience will have positive outcomes on healthy weight [PM11] |
|  | Very effective, I would say, because it's quite a status to kids having burgers, to be able to walk around the road showing to their friends that haven't got a burger, "Oh, I've got a burger." You know what kids are like, it becomes a big thing, it's not just a burger anymore, it's a status symbol. [P9, F/60-79/Intermediate/White] | Things like the green space policies, trying to encourage again perhaps, like, growing spaces, thinking of food production and strengthening local food economies to champion the local supply chains. And, again, this is a bigger topic than just purely healthy weight and diet, but it could certainly be one of the outcomes if you build those considerations into town or city planning [PM12] |
|  | I think there should be maximum working hours anyway. I don’t think it necessarily gives more time for people to exercise and prepare food, there just should be a reduction… well, not necessarily a reduction, but making sure that people work reasonable working weeks and not these silly hours [P1, M/60-79/High/White] |  |
|  | I think they could be really effective. The tax break, I mean I always think that’s the answer; money. And if you’re a government and you look at it as a tax break and therefore it’s going to look to them, everyday man saving some money, then the bigger that is the better, and the more likely you’ll be to do it. [P10, M/40-59/High/White] |  |
| - Valued co-benefits | it’s reducing congestion on the roads, isn’t it? Because the parents are not getting in their 4x4 and taking the kids to school every day, so yes, it’s a definite plus. [P8, M/40-59/Intermediate/White] | And that is very much about air quality and it is about improving air quality and improving lifestyle and improving the general health of people within the areas where we have the greatest congestion. [PM13] |
|  | Going back to congestion charge, yes, anything that cuts down CO2 in a city is a good thing [P11, M/60-79/High/White] | I do firmly believe that the, sort of, town and city planning policies are the, sort of, way forward because of what I said in terms of, you know, people don't know then that they're the subject of an active travel policy; they are just living in a better environment. [PM12] |
|  | Sugar taxes, I think the idea of cutting down on sugar within sweets and drinks, all that kind of thing, I don’t have a problem with that. I think that’s quite a good idea. It’s also a useful form of revenue for the government, isn’t it? [P1, M/60-79/High/White] | I think some of those campaigns are good because it’s more about healthy choices, being healthy and being more active. Often, they’re combined with that will help your mental health as well and your relationships as well as your physical health. It isn’t all focused on weight loss. [PM4] |
|  | Just in the next town to where I live they’ve just started all these bicycle lanes. What it’s done is actually created an even bigger problem. Where there’s a normal road, they’ve actually reduced the size of that to basically put in these cycle lanes. […] It’s creating more congestion, [P18, M/40-59/High/ Asian Indian] |  |
|  | I think there should be maximum working hours anyway. I don’t think it necessarily gives more time for people to exercise and prepare food, there just should be a reduction… well, not necessarily a reduction, but making sure that people work reasonable working weeks and not these silly hours [P1, M/60-79/High/White] |  |
| - Focus on young people | I'm all for it, [interviewer name], I think it’s a great idea [inform and educate policies]. I think it should be, it needs to be, the young people need to be educated because then obviously that just then rolls forward. When they start getting older then their children will be better fed and I'm all for educating the young ones in a healthier way of living. [P14, F/40-59/Intermediate/White] | There’s a big focus on prevention. When you’re looking at prevention, the earlier you can get to people the better. Some of the problem we have in adult services is by the time people come to us needing support and asking for help, they’ve already gone down a route that makes it really difficult for them to change, for whatever reason. [PM4] |
|  | Yes, I would support it more because I feel like if we tackle children from young, then you'd make better, healthier adults. So, if you've got a child who was able to cycle or enjoyed riding then maybe when they got older they would then be someone who cycled to work. [P16, F/20-39/Intermediate/Black] | I think if we really focused some of our energy on teaching young people to be healthier adults, we would have a healthier population. [PM16] |
|  | The thought of going out and playing football or something is probably not on their high list of things. […] They just get into a couch potato scenario where that’s all they want to do, they just want to sit in front of the thing and play games. Rather than being active and actually doing things. [P18, M/40-59/High/ Asian Indian] | So childhood obesity is a massive focus for us. Obviously we’ve got childhood obesity plans, declaration, tracking health inequalities, is a massive focus for us. But also adults […] But if we don’t respond to adults who are already kind of getting Diabetes, having worst outcomes around cancer and CBD, then we’re failing the population completely, so we have to do all of it [PM10] |
|  | Well I think everybody needs to lose weight, so it shouldn’t be just down to children only, should it? Why have cheaper veg and fruit for young people when older people are overweight? [P19, M/40-59/Low/White] |  |
|  | Well convenience foods are not good for children, are not good for anybody, sweets are not good for anybody. It doesn’t matter what age you’re going at, it doesn’t matter, it’s the same answer irrespective of the age of the consumer. [P2, M/60-79/Intermediate/White] |  |
| Barriers | ‘There’s all that advertising and media and stuff that goes with [non-nutritious food]. But also getting down to the price, it’s cheaper to buy a box of cornflakes […] [P4, F/40-59/High/Black] | Tax breaks for bikes and buses, I think more people would use public transport if it was cheaper than getting in their car, but it's currently not. [PM16] |
|  | if I could have a tax break from using my bike, which I think I know this already that I can, then I would. But like I said to you, if I got wet, I would be less likely to do it. [P10, M/40-59/High/White] | We know there are a lot of families, particularly locally, that would be very eligible for going to a food bank and getting lots of food provision, but the stigma of going there stops them going. [PM3] |
|  | Then, it needs to be actively policed as well, people parking in cycle lanes, people undertaking in cycle lanes, you know, that goes on all the time. If you’re seeing that every morning, it would put you off letting your kids get on a bike and riding to school. [P12, M/40-59/Low/White] | when you look what the Dutch have achieved with their infrastructure, the Dutch people don’t perceive they have to be encouraged to go out and cycle because it’s just normal. [PM8] |
|  | Bus commuting, that’s also impractical because we haven’t got the structure. It’s so hard to mix general traffic and bus traffic. [P2, M/60-79/Intermediate/White] | It is not just […], “Can you be part of a cycle to work scheme?” It is, “Can you afford a bike? Can you afford the time to cycle to and from work? Do you live in an area where it safe to do so? Are you allowed to have access to showers and lockers when you actually get to your job? Is it socially acceptable in your peer group of colleagues? [PM5] |
|  | It’s just that society we live in where we feel we’ve got no time. I think that mindset needs to probably change as well to spend some time cooking, to plan your meals and stuff like that. Rather than to say I’m choosing the easy way out, let’s go and get a Domino’s. I think it’s changing people’s mindset. [P18, M/40-59/High/ Asian Indian] |  |
|  | The wife’s over listening, and she’s put price to eat well… To eat well it costs more, she’s saying. So if you want fresh fruit and fresh veg in your evening meal, then it’s going to cost you a lot more than a frozen lasagne for four people. [P19, M/40-59/Low/White] |  |
| **Theme 2: Perceived policy fairness** | | |
| Opportunity to benefit | Yes, of course, I would say definitely. We were offering everybody the tools they need to try and make some healthy choices; that’s fair to everyone. [P10, M/40-59/High/White] | N/A |
|  | I do think that one is fair to all people because you’re not really taking much away to build a cycle lane, maybe a bit of grass or something like that, so I think that would… Providing more routes of access to get places more directly, I think everyone would gain from that. [P6, F/20-39/Intermediate/White] | N/A |
| Equality | The congestion charge is a bit harsh. Because if you work in the centre of [city name], then you’re going to have to pay it, aren’t you? You haven’t got any choice unless you move jobs. So that is a little bit unfair. [P19, M/40-59/Low/White] | I think that these [inform and educate policies] will affect- of all of the areas, will probably be the least equal in its impact, in terms of, I think we'll end up targeting the same group of people who aren't the highest risk. [PM11] |
|  | It applies to everybody whatever background you come from [C4L campaign]. It’s fair to everybody, yes. [P18, M/40-59/High/ Asian Indian] | Often, it feels like [Change4Life is] aimed at people who already have some agency and some understanding of these kinds of things and are interested in it already because most people who aren’t will just ignore it. It’s about us trying to make sure we’re not widening inequalities gaps again by giving more information to people who’ve got the support system around them to make healthier choices. [PM4] |
|  | I think on these sort of ones it does. I think almost regardless of status, most people can go with a bike and use that, and if they can’t, it’s normally cycle lanes; it’s a cycle lane, but it’s a walkway as well. So, I think that’s pretty even. [P8, M/40-59/Intermediate/White] | One assumes that the council would have the ability to make exemptions, for example disabled people who have no alternative but to drive. One assumes that an exemption would be made for them, otherwise it wouldn’t be fair. [PM17] |
|  | Preventing takeaways building, that’s unfair. That’s unfair to the takeaway guy that wants to open his shop [P2, M/60-79/Intermediate/White] | The Change4Life one, yes. I mean, if people don’t have access to the internet or a smartphone, maybe not, because a lot of their apps and useful tools are on there. So, in an extreme scenario, then maybe not as equal as we think it might be. [PM18] |
|  | In regard to the congestion charge, like I said, having mobility issues myself, as well as the Cycle to Work scheme, that’s something, especially in the winter, I wouldn’t be able to do. [P20, F/20-39/Intermediate/Mixed] |  |
|  | And then the active travel one, well that might be unfair to people that live outside of the area they work in, who don’t have the chance to cycle to work. [P10, M/40-59/High/White] |  |
|  | Reasonably saying that it would be unfair to those people that want a larger portion of convenience food, that don’t want plain packaging, so it’s unfair, of course it’s unfair. It might be right but, once again, that doesn’t mean it’s fair. [P2, M/60-79/Intermediate/White] |  |
| Rewarding vs. penalising behaviour | I think subsidising healthier choices would perhaps be more beneficial, encourage people rather than punish them almost [P5, F/20-39/Intermediate/White] | What I do really like is things like subsidies for fruit and vegetables. Rather than being punitive on the other things, being able to subsidise fruit and vegetables and give people free food or low-cost food that’s healthy. [PM4] |
|  | I think, for example with Tesco which I’ve got my Clubcard with, it’s something like you get a point for every pound that you spend. Instead of every pound that you spend it should be, maybe, you get two points for each healthy item you’ve bought or what’s classed as healthy. Something along those lines where you’re incentivising and rewarding for the healthier option rather than taking the unhealthy one away, I think, is probably the way to go [P6, F/20-39/Intermediate/White] | the subsidies for fruit and vegetables, the cycle to work scheme and tax breaks for bike and bus commuting, those ones are all ones where you get a benefit for doing the right thing. So, they are completely acceptable.[PM17] |
|  | Like maybe, say on the convenient food, why not add some spinach with that or a side salad, to get one of your five-a-day? Or, did you know splitting this portion, you can make a meal for two by adding-? I don't know. I just don’t think you should just reduce the portion. [P20, F/20-39/Intermediate/Mixed] | if that was balanced with reducing portion size of convenience foods and providing more access, cheaper access to non-convenience foods, great. But, at the moment, that's just a negative that's taking something away, which I think is where you then get negative connotations from the public. [PM3] |
| **Theme 3: Communication of policies** | | |
| Visibility of policies | It’s also how you word it, how you frame it, how you present it, that this is a good thing to do. I’m sure there are lots of examples of ways to make cycling to work or walking to work attractive to the employees. [P4, F/40-59/High/Black] | If you make infrastructure changes, how you communicate those, and how you support and enable people to benefit from them. [PM10] |
|  | it’s a name that I know [C4L], but I couldn’t even tell you what it did. It might say the name says it all; it really doesn’t. That could be a heart disease foundation, couldn’t it? I don’t think that was particularly successful. [P10, M/40-59/High/White] | We had some government money for energy efficiency measures. So people could, you know, have grants for insulating their homes. […]  We publicise that through all the measures we could and still people didn't, sort of, come forward. Now that might well be for other reasons that they didn't want the upheaval and stuff, and I know it doesn't apply to, sort of, active travel, which is what we're talking about, but it just sort of highlights how difficult it is even when you're doing something that you see as really positive to, sort of, give something away. [PM12] |
|  | I just don't feel like it's as well-known as it should be, I feel like if I remember rightly, they used to have advertisements on the TV [P16, F/20-39/Intermediate/Black] | I'm not surprised they do think they’re more effective, because [town and city planning policies are] visible things that you can see that has actually happened. That makes a difference. So, I can see why compared to the economic approach, which is more of an invisible intervention, there is something culturally about, you can see they’re putting a mark in the sand about that. [PM5] |
|  | I think they could be effective, but I think they need to be more embedded in- For instance, I got that information from my health visitor, I haven’t seen my health visitor in two years now. So, where I would ask that information- I guess if I googled it. But there is nothing prompting me to google it. [P20, F/20-39/Intermediate/Mixed] |  |
| The role of media | N/A | I think one of the big influencers for the population around policies- some of them are quite media-driven, aren’t they? So it can be really what’s out in the news at the time, the portrayal of that that comes across, in terms of how engaged people are when using them, or engaging with them. [PM3] |
|  | N/A | the Change4Life campaign is something I'm more newly aware of because of the area that I'm now working in. I think it's absolutely fantastic, but actually I knew absolutely nothing about it before. Nobody I know knows anything about it before. [PM16] |
|  |  | the sugary drinks tax attracted a lot of press about the disproportionate impact on low-income people, even though it technically shouldn't, if it's a reformulation, but you know, that's, kind of, press coverage, it attracts. [PM11] |
| **Theme 4: Improving policy support** | | |
| Improving policy support: perceived policy effectiveness | ‘I think we need to find a way to make healthier food cheaper, but we need to ensure that is also alongside an access piece, and an education piece as well’ [P1, M/60-79/High/White] | Obviously, I think it is important also that we recognise all of these policies, as individual policies, they are not going to be the answer. We have got to act at every single level [PM1] |
|  | If you get a safe space for people to cycle on, they can all go, a little family bike ride and things like that, as long as there were bikes available and affordable for people to use. They go hand in hand with the other cycle scheme maybe. [P14, F/40-59/Intermediate/White] | subsidies or financial aid for fruit and vegetables, I think it's wonderful, but it needs to go hand in hand with learning, and of cooking skills and that kind of stuff, to enable them to take advantage of that fruit and veg. [PM15] |
|  | I think it's a good idea. I do think it's a good idea to keep the cars off the road, but then my thing is, "Well, yes, keep the cars off the road, but give us a decent bus service." [P13, F/60-79/Low/White] | But I do think there needs to be that little bit of education around it. It’s all well and good having red, orange and green on the front of the pack, but there are some foods which don’t necessarily follow the rules. there is red for sugar on fruit, but people might think, “Oh, this packet of crisps is green for sugar, so maybe I should eat that.” [PM18] |
|  | The congestion charge sort of area, you know, lots of that obviously makes sense, but I think the way to improve that is to make something that is a realistic price, and I mean realistic. [P10, M/40-59/High/White] |  |
|  | Yes. I mean, it’s hard to direct it in one language, isn’t it, but, then, all of a sudden, put it in all languages, so people can do it? I think if it’s more, sort of, visual, people can take that information away without even understanding the language. Do you know what I mean? [P12, M/40-59/Low/White] |  |
| Improving policy support: perceived policy fairness | The whole generation, it’s like these policies are great for those who are working and stuff [Cycle2Work], but it needs to be across the board for people to be encouraged to get out through the day time and go for a walk or whatever. [P14, F/40-59/Intermediate/White] | I don’t think anyone’s disadvantaged by that. As long as what you’re doing in terms of working policies are the same as people who are not in work. You know, that you’re actually enabling and supporting people who are- If you are enabling people and supporting people who are in work to have a better access to being able to exercise and eat well, then you need to do the same for people who aren’t in work, I guess. [PM10] |
|  | hose you mentioned who are maybe disabled or poor, we need to cater for those as well. There has to be schemes where it’s more affordable for them to travel, like free travel for people who are on low incomes and things like that, so it encourages them to use public transport. [P18, M/40-59/High/ Asian Indian] | for example disabled people who have no alternative but to drive. One assumes that an exemption would be made for them, otherwise it wouldn’t be fair. [PM17] |
|  | So, as I say, having a display of fruit and veg at the checkout I think is more positive, you're encouraging people to make the right choice rather than taking away free choice. [P5, F/20-39/Intermediate/White] | I think our role is to understand that policy, but also look to implement it so that you can almost share the benefits of that across those different groups. So, actually, they can see that improvement in the use of cycle lanes, and the accessibility of cycle lanes in urban and rural areas, benefits a much wider cohort, than just the people that might use the cycle lanes, climate change argument, that kind of thing again. [PM3] |
|  | We need education, more… It’s got to be education, as opposed to policies that stop you choosing. [P2, M/60-79/Intermediate/White] |  |
| Improving policy support: communication of policies | I think the general way to go […] is not taking away from the general public but incentivising the healthier options […] and giving them a reward that actually matters […] like a percentage off their food bill’ [P6, F/20-39/Intermediate/White] | I think that consultation and communication about changes is really critical, and I think involving the community in local community based changes and development is really, really absolutely critical in terms of, you know, community engagement and buy-in to change it. [PM10] |
|  | ‘I guess the important thing is […] where those taxes end up, do they end up back with the health industry, to support NHS […]? Yes, I think if you knew that, […] then absolutely, that would definitely swing decisions’ [P8, M/40-59/Intermediate/White] | So, I don’t think it’s deliberate. But I do think including people and letting them know, having that transparency, would definitely help, in a way. [PM18] |
|  |  | It probably comes back to the access piece. The local population are flooded with unhealthy associated behaviours, so I think all of these around informing and educating, really need to be going at a scale that people are seeing more and more consistent messages that align to healthy eating, active travel. [PM3] |
